# Infant Care: Predictors of Outdoor Walking, Infant Carrying and Infant Outdoor Sleeping

**DOI:** 10.3390/ijerph21060694

**Published:** 2024-05-28

**Authors:** Nicole Rheinheimer, Stefania V. Vacaru, Julie C. van Immerseel, Simone Kühn, Carolina de Weerth

**Affiliations:** 1 Donders Institute for Brain, Cognition and Behaviour, Radboud University Medical Center, 6525 GA Nijmegen, The Netherlands; 2 Department of Psychology, New York University-Abu Dhabi, Abu Dhabi P.O. Box 129188, United Arab Emirates; 3 Department of Clinical Child and Family Studies & Amsterdam Public Health, Vrije Universiteit Amsterdam, 1081 HV Amsterdam, The Netherlands; 4 Center for Environmental Neuroscience, Max Planck Institute for Human Development, 14195 Berlin, Germany; 5 Department of Psychiatry and Psychotherapy, University Medical Center Hamburg-Eppendorf, 20249 Hamburg, Germany

**Keywords:** infancy, childhood, outdoor activities, pram walking, infant carrying, sleep, demographic correlates, environment

## Abstract

Background. Although spending time outdoors is beneficial for development, little is known about outdoor time during infancy. The aim of this study was to assess frequencies and durations of (1a) outdoor walking and carrying in mother–infant dyads and (1b) infant outdoor sleeping in a stationary cot or pram. We furthermore aimed to identify associations of (2a) outdoor walking and carrying and (2b) infant outdoor sleeping, with infant, maternal and environmental sample characteristics. Methods. An online survey was distributed among mothers of 0- to 12-month-old infants. Initially, 1453 mothers were recruited, of which 1275 were included in the analyses. With respect to (1a) the outcomes of interest were: mother–infant dyads’ total weekly duration of walking in minutes, frequency of walking on weekdays, as well as weekends, and the frequency of using an infant carrier during walks, as well as the daily duration of carrying in hours (indoors and outdoors together). With respect to (1b) the outcome variables were: placing the infant outdoors to sleep (yes/no), the total weekly duration of outdoor sleeping and the weekly frequency of outdoor sleeping. For aim 2, associations of the outcome variables with infant (i.e., age), maternal (i.e., working status) and environmental (i.e., house type) sample characteristics were assessed. Results. Mother–infant dyads engaged in walks for a total weekly duration of 201 min, for approximately one to three walks over weekdays (Monday through Friday), as well as one to three walks on the weekend. The infant carrier was used by 22% of mothers at least half of the time during outdoor walks, and 18% reported a daily duration of infant carrying of one hour or more. Among other associations, infant and maternal enjoyment of outdoor walking correlated positively with the duration as well as the frequency of walking during weekdays and during the weekend. Furthermore, employed mothers walked for a shorter duration and less frequently on weekdays as compared to mothers on maternity leave or mothers without a paid job. The availability of nearby recreational areas correlated positively with the weekly duration and frequency of walks. The infant carrier was used more frequently during outdoor walks if more than one child lived in the household. Infant carrying during outdoor walks was also related to infant behavior at night. Roughly a third of the mothers (29%) regularly had their infant sleep outdoors for a weekly duration of four hours and a weekly frequency of approximately one to two times. Younger infants, infants of mothers with higher education and infants living in detached houses were more likely to be placed outdoors to sleep. Discussion. We identified associations of infant, maternal and environmental characteristics with outdoor time spent during infancy. These results lay the foundation for future research on the effects of the outdoors on child development as well as on facilitators and barriers for caregivers.

## 1. Introduction

The first year of life characterizes a sensitive period for a multitude of developmental processes, and factors in the early caregiving environment have been related to longitudinal outcomes of physical and mental health [1,2,3,4]. One factor that has been demonstrated to benefit child health and socio-emotional regulation is exposure to the outdoors [5,6,7,8,9]. In the past decades, however, there has been a downward trend in the time children spend outdoors around the globe, and nowadays, children from infancy to early adolescence spend less than 15% of their wake-time outdoors [6,10,11,12]. Identifying demographic characteristics that might facilitate or hinder outdoor time during infancy can deliver crucial insights for urban planning and caregiving advice, as well as for policies and interventions to facilitate outdoor exposure during infancy. Research on older children indicates that time spent outdoors depends on a number of child-specific characteristics, such as age and sex, parental characteristics, including socio-economic and employment status, as well as the living environment [7,11,13,14,15].

While studies in older children focus largely on active outdoor play [16,17,18], time spent outdoors during infancy is more passive, as infants need to be taken outdoors by their caregiver, for instance, on a walk using a pram or carrier, or to sleep outdoors in a stationary cot. To date, there is a lack of studies on the frequency and duration of these outdoor activities during infancy and the demographic characteristics that might be associated with these activities. This study aimed to assess frequencies and durations of outdoor walking and carrying in mother–infant dyads, as well as infant outdoor sleeping in a stationary cot or pram, and to identify associations of these activities with infant, maternal and environmental sample characteristics.

Infancy is a sensitive period of dramatic and rapid developmental processes, involving the immune system, brain development, gut microbiota, thermo-regulation and the stress system [2,3,4,19]. During this period, outdoor exposure might be especially beneficial, as research in older children indicates that outdoor exposure is associated with decreased risk for myopia [6,20], increased vitamin D levels [21] and improved mental health [7,22], as well as cognitive and socio-emotional development [8,9,23,24,25]. Furthermore, a study on young adults found positive associations of time spent outdoors in the past 24 h with mood and gray matter volume in the brain, also after accounting for physical activity, intake of fluids, amount of spare time and the hours of sunshine [26]. Accordingly, a study assessing the area surrounding children’s residential address from birth until age 12 found positive associations of the visibility of the sky and the amount of open green space, as well as negative associations of tree cover density, with gray matter volume in areas of the brain at age 12 [27]. Another study on the residential environment found that more greenness throughout childhood was positively correlated with both gray and white matter volume at primary school age [28]. Previous studies indicate that especially the amount of outdoor greenspace might play a role in the positive effects of the outdoors on child development. However, to date, the underlying mechanisms of the effect of the outdoors on child development are largely unknown.

Researchers suggest that the outdoors provides children with opportunities to observe, learn about and interact with their surroundings, thereby facilitating brain development [29,30]. Notably, most studies on benefits of the outdoors for child development focused on older children, who spend most of their outdoor time on active play, which may account for the beneficial effects of outdoor exposure, for instance, due to increased physical exercise [16,17,18]. During infancy, outdoor time is likely to be more passive, as infants rely on caregivers to take them outdoors, for instance, on a walk using a pram. While potential benefits of outdoor walking for infants have not been studied, pram walking has been shown to decrease postnatal depression in mothers [31].

Furthermore, infants highly depend on proximity and responsive interactions with their caregivers [32,33]. A mode of transporting the infant in close proximity during a walk is infant carrying, by wearing a carrier or sling on the chest or on the back. Developmentalists suggest that increased proximity through infant carrying fosters an exchange of sensory cues and increases maternal responsiveness to infant vocalizations, which, in turn, is suggested to facilitate the development of stress regulation capacities [32,33,34,35]. While the benefits of infant carrying in the outdoors have not been studied to date, there is evidence of a calming effect of being carried on the cardiovascular stress response in a laboratory setting [36], and regular infant carrying has been associated with improved mother–infant bonding [33,37]. Furthermore, especially in Scandinavian countries, from the age of two weeks, infants are frequently placed outdoors to sleep in a stationary cot or pram in a garden or on a terrace or balcony, and this has been related to increased sleep durations [38]. While infant outdoor sleeping has been reported to be common in Scandinavia, and is also regarded as safe in cold winters [39], no studies to date have assessed this practice in other, more temperate climate regions of Europe, and hence the prevalence is unknown to date.

Considering the potential benefits of outdoor exposure for child development, promoting outdoor activities during infancy is a promising avenue for intervention. In order to promote healthy behavior in society, the behavioral epidemiology framework states that it is crucial to first identify demographic characteristics predicting the behavior [40]. To date, there is a lack of studies on how much time infants spend on outdoor activities, such as being walked or sleeping outdoors. One study found that the greatest barrier for mothers to walk outdoors with a pram were undesirable weather conditions, neighborhood walkability, as well as a lack of time, however, this study was restricted to maternal opinions regarding postnatal exercise in the outdoors [41].

While there is a lack of research on outdoor activities during infancy, a large number of studies have identified child-specific, parental and environmental characteristics predicting outdoor time in older children [14,15,42]. For instance, studies report less outdoor time in girls, in children of older age, and in children of mothers who are employed and have a higher education level [42,43,44,45]. Children spend more time outdoors when living in detached houses, when the neighborhood is more rural or is perceived as more safe and when there are more recreational areas around, such as parks and playgrounds [12,45,46,47,48]. Furthermore, less outdoor time was reported in colder seasons [12,49], and especially in the past years, the COVID-19 pandemic has imposed temporary restrictions on children’s time outdoors [50,51].

The aim of this study was to assess (1a) the frequency and duration of outdoor walking and carrying in mother–infant dyads and (1b) the frequency and duration of infant outdoor sleeping in a stationary cot or pram in the garden or on the balcony or terrace. Aim (2) was to identify associations of (2a) outdoor walking and carrying and (2b) infant outdoor sleeping with several factors. These factors included infant characteristics: age, sex, gestational age at birth, preterm birth, having health issues, infant behavior at night and whether the infant enjoyed being walked outdoors; maternal characteristics: age, education level, employment status, working hours, having mental or physiological health issues and whether the mother enjoyed walking outdoors with the infant; and environmental characteristics: city size, availability of nearby recreational areas, housing type, number of children and adults in the household and season. The study was based on data obtained with an online survey among mothers of infants in the Netherlands.

## 2. Methods

### 2.1. Recruitment and Participants

The ethics committee of the Faculty of Social Sciences of the Radboud University Nijmegen reviewed the study and did not have formal objections (SW2017-1303-497). The study was in accordance with the Declaration of Helsinki and preregistered (https://aspredicted.org/NTC_MJQ, accessed on 19 April 2023). Recruitment was performed in the Dutch language and took place online between April 2022 and April 2023 through social media, using paid ads on Instagram and Facebook (targeting mothers residing in the Netherlands), printed flyers at the Baby and Child Research Center Nijmegen (BRC) and a participant database of the BRC including mothers in the Netherlands interested in research participation. Although participation from outside of the Netherlands was not ruled out, given the recruitment strategies, it is likely that the majority of mothers resided in the Netherlands during participation. Inclusion criteria were: Dutch fluency, maternal age > 18 years, infant age < 53 weeks and the infant not being a twin.

In line with the exploratory and descriptive nature of this study, determining a maximum sample size was not of interest and, therefore, a priori power calculations were not required [52]. The sample size depended on the budget available for recruitment and on recruitment continuing for a year to cover all seasons. In total, 1453 participants were recruited. From these, 176 participants were excluded, as they did not complete the survey until the first outcome variable ‘frequency of walking on weekdays’ (<49% of all items), and 2 were excluded due to providing illogical answers. Binomial logistic regressions indicated that excluded (*N* = 176) and included (*N* = 1275) participants did not differ in maternal and infant age, maternal education or whether infant or maternal health issues were reported (*p* > 0.48).

### 2.2. Procedure

Mothers provided informed consent and filled in an online survey (46 items; average duration of 9 min). After survey completion, mothers could indicate whether they would like to participate in a draw to win a gift voucher worth EUR 50 with a chance of 1 in 50. The draw was performed by creating 1 random number for every 50 participants using the ‘runif’ function in R [53] and a total of 21 gift vouchers were distributed.

### 2.3. Measures

Due to a lack of existing tools and literature on outdoor time of mothers and infants, the survey was developed by the authors through repeated research group meetings and piloted among colleagues with infants. The complete survey can be found in the Supplementary Materials (S1). Table 1 provides a descriptive summary of all study variables.

#### 2.3.1. Outdoor Walking and Carrying

Mothers were asked the following questions on outdoor walking with their infant: their total weekly duration of walking in minutes with their infant and the frequency of walking with the infant on weekdays (Monday through Friday), as well as on the weekend (Saturday and Sunday), by counting all walks with a duration of at least 15 consecutive minutes. Mothers were additionally asked whether they were satisfied with the amount of walking with the infant and which subjective reasons for and against walking applied to them. Infant carrying was assessed with the following variables: the frequency of the mother using an infant carrier during outdoor walks, as well as the total daily duration of the mother carrying the infant, regardless of whether it was indoors or outdoors.

#### 2.3.2. Infant Outdoor Sleeping

Outdoor sleeping was defined as the infant being placed outdoors in a stationary cot or pram (i.e., garden, terrace or balcony) for a nap. We did not distinguish between outdoor sleeping at home or elsewhere (e.g., at daycare) or whether it was performed by the mother or another caregiver. Outdoor sleeping was assessed with the following variables: whether the infant was placed outdoors to sleep at all (yes/no), and if the answer was ‘yes’, the total weekly duration and the weekly frequency of outdoor sleeping.

#### 2.3.3. Infant, Maternal and Environmental Sample Characteristics

The following infant characteristics were collected: age in weeks, sex, gestational age at birth in weeks, preterm birth (<37 gestational weeks), having one or more health issues, infant behavior at night (how much attention the infant needs at night, how much difficulty the infant has falling asleep and how often the infant wakes up at night) and whether the infant enjoyed being walked outdoors. Maternal characteristics collected were: age in years, education level, employment status, weekly working hours, having one or more mental health issues, having physiological health issues and whether the mother enjoyed walking outdoors with the infant. Environmental characteristics collected were: city size, sum of different types of recreational areas nearby (in walking distance), housing type, having more than one child in the household, having more than one adult in the household and the season during participation.

### 2.4. Analytical Plan

Statistical analyses were performed in R [53] using the following packages: ggplot2 [54], ggstatsplot [55], rcompanion [56], car [57], aod [58], Boruta [59], psych [60], stats [53] and rstatix [61].

#### 2.4.1. Preliminary Analyses

The data were visually inspected and cleaned. Errors that were clearly mistakes and typos (e.g., unrealistic gestational age at birth) were replaced with missing values. Outliers on continuous outcome variables (scores greater than three times the standard deviation above or below the mean) were winsorized [62]. Skewed continuous outcome variables were square root transformed for the main analyses. Missing data were not imputed.

#### 2.4.2. Main Analyses

##### Aim 1: Assessing the Frequency and Duration of Outdoor Walking and Carrying in Mother–Infant Dyads, as Well as of Infant Outdoor Sleeping

For aims (1a) and (1b), we calculated descriptive statistics. For continuous outcome variables, means, standard deviations and ranges were computed. Categorical and ordinal variables were summarized by computing frequencies and percentages per response category.

##### Aim 2: Identifying Associations of Outdoor Walking and Carrying, as Well as Infant Outdoor Sleeping, with Infant, Maternal and Environmental Characteristics

If both the predictor and the outcome variable were continuous and normally distributed, Pearson correlations were performed, and if one of the two variables was ordinal or non-normally distributed, Spearman correlations were used. Differences for a categorical predictor on a continuous outcome variable were assessed using Student’s *t*-tests for normally distributed data and Mann–Whitney U tests for non-normally distributed data. More than two groups were compared using analyses of variance for normally distributed data and Kruskal–Wallis tests for non-normally distributed data. If both the predictor and the outcome variable were categorical, chi-square tests were used, and associations of continuous predictors with categorical outcome variables were assessed using binomial logistic regressions. The corresponding tables in Section 3 display which statistical test was performed per analysis. Statistical significance was based on *p*-values. As we performed separate analyses for a large number of predictors (*N* = 154), the significance level was corrected for multiple testing through the Benjamini–Hochberg procedure [63] using a false discovery rate of 10%.

Additionally, for our main variables of interest, we used the Boruta algorithm in an exploratory manner [59] to assess the importance of all infant, maternal and environmental characteristics in predicting the following outcome variables: total weekly duration of walking, frequency of using an infant carrier during outdoor walks and whether the infant is placed outdoors to sleep at all (yes/no). Boruta is a powerful feature selection algorithm, offering a comprehensive overview of all variables relevant in predicting a response variable, regardless of multicollinearity and non-linear relationships between variables, and hence has the potential to deliver informative insights for future research [64]. As such, this Boruta algorithm complemented our preregistered analyses aimed at discovering factors associated with outdoor activities with the infant. As a wrapper around a random forest algorithm, Boruta creates importance scores for each variable and compares these scores to that of randomly permuted so-called ‘shadow’ variables. Variables receiving an importance score significantly higher than all shadow variables are labeled ‘important’, while items receiving significantly lower importance scores are labeled ‘unimportant’. The algorithm stops when all variables are labeled or when a maximum of 20,000 predefined iterations has been reached.

## 3. Results

### 3.1. Preliminary Analyses

The following unrealistic values were replaced with missing values: gestational age at birth below 22 weeks (*N* = 2), as live births before week 22 are rare, and total weekly duration of outdoor sleeping of more than 42 h (*N* = 21), as infants commonly nap for up to 6 h during daytime [65]. The following outliers (scores greater than three times the standard deviation above or below the mean) were winsorized [62]: total weekly duration of walking (*N* = 24) and total weekly duration of outdoor sleeping (*N* = 8). The variable ‘total weekly duration of outdoor walking’ was negatively skewed and hence square root transformed so that normality was achieved.

### 3.2. Main Analyses

#### 3.2.1. Aim 1: Assessing the Frequency and Duration of Outdoor Walking and Carrying in Mother–Infant Dyads, as Well as of Infant Outdoor Sleeping

Overall, mothers reported walking outdoors with the infant for approximately 201 min weekly (*SD* = 170). On average, mothers reported walking outdoors with their infant between one to three times throughout the week (Monday to Friday) and one to three times on the weekend (Saturday and Sunday). When walking outdoors, 22% of mothers used an infant carrier half of the time or more. Overall, 29% of infants were placed outdoors to sleep, for a mean of 4.31 h a week (*SD* = 5.27), and approximately one to two times a week. Table 1 displays the descriptive statistics for the outdoor variables, as well as for the infant, maternal and environmental characteristics.

#### 3.2.2. Aim 2: Identifying Associations of Outdoor Walking and Carrying, as Well as of Infant Outdoor Sleeping, with Infant, Maternal and Environmental Characteristics

All significant results on the associations of the sample characteristics with the outcome variables after the Benjamini–Hochberg correction for multiple testing are summarized in the following sections. A complete overview of the Benjamini–Hochberg corrections can be found in the Supplementary Materials (S2).

##### Outdoor Walking and Carrying

All results of the analyses on the associations between infant, maternal and environmental characteristics and the outcome variables are reported in Table 2.

1. Infant Characteristics

For infants with a greater enjoyment of being walked, we found a longer weekly duration of walking (correlation coefficient *r*(degrees of freedom = 1207) = 0.22, *p* = 0.000, 95%CI [0.16, 0.27]), as well as a higher frequency of walking on weekdays (*r*(1207) = 0.18, *p* = 0.000, 95%CI [0.13, 0.24]) and on weekends (*r*(1207) = 0.22, *p* = 0.000, 95%CI [0.16, 0.27]). For younger infants, we found a higher frequency of walking on weekdays (*r*(1273) = −0.08, *p* = 0.004, 95%CI [−0.13, −0.02]). We found a longer weekly duration of walking (*t*(1225) = −2.30, *d* = 0.23, *p* = 0.022, 95% CI [−2.77, −0.22]) in preterm infants (*M_minutes_* = 236.13, *SD* = 156.42) as compared to infants born full-term (*M_minutes_* = 194.96, *SD* = 154.08). A lower gestational age at birth was associated with a higher frequency of walking on weekdays (*r*(1271) = −0.07, *p* = 0.018, 95%CI [−0.12, −0.01]). We found a positive correlation of infant behavior at night with the daily duration of carrying (*r*(1171) = 0.20, *p* = 0.000, 95%CI [0.14, 0.25]), as well as with the frequency of using the infant carrier during outdoor walks (*r*(1179) = 0.16, *p* = 0.000, 95%CI [0.10, 0.22]). For younger infants, we also found a longer daily duration of carrying (*r*(1171) = −0.14, *p* = 0.000, 95%CI [−0.19, −0.08]).

2. Maternal Characteristics

**Figure 1:**
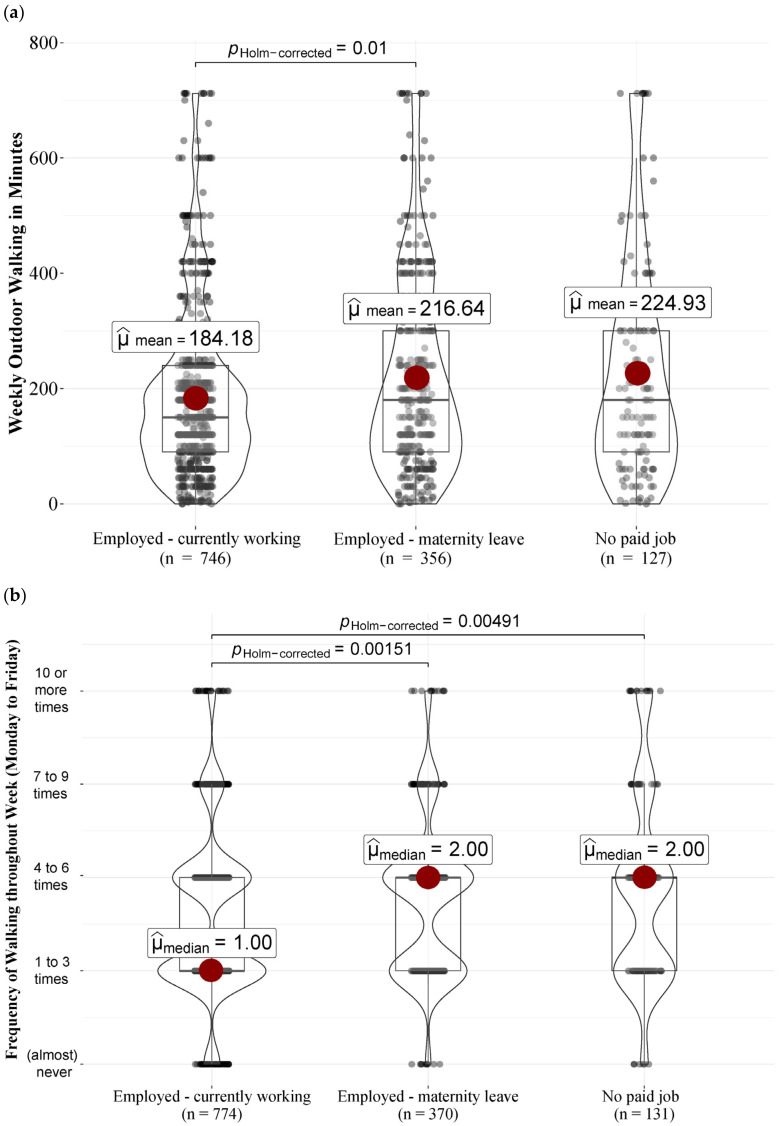
Violin plots on the association of employment status with (**a**) weekly duration of outdoor walking in minutes and (**b**) frequency of outdoor walking on weekdays (Monday to Friday). The width of the violin shape represents the distribution of the data, with a larger width indicating a higher frequency of scores. The red dot indicates the group mean (**a**) or median (**b**).

Greater maternal enjoyment of outdoor walks was related to a longer weekly duration of walking (*r*(1210) = 0.24, *p* = 0.000, 95%CI [0.18, 0.29]), as well as higher frequencies of walking on weekdays (*r*(1210) = 0.20, *p* = 0.000, 95%CI [0.14, 0.25]) and on weekends (*r*(1210) = 0.23, *p* = 0.000, 95%CI [0.18, 0.29]). There was also a difference for maternal education in the frequency of walking on weekdays (*U* = 188,846, *N* = 1275, *r* = 0.13, *p* = 0.000) and on weekends (*U* = 174,236, *N* = 1271, *r* = 0.08, *p* = 0.004). Higher-educated mothers reported walking approximately ‘one to three times’ (*Mdn* = 1, *M* = 1.57) with their infant on weekdays, while lower-educated mothers reported walking ‘four to six times’ (*Mdn* = 2, *M* = 1.83). Likewise, on weekends, higher-educated mothers (*Mdn* = 1, stands for ‘one to three times’, *M* = 1.10) reported a slightly lower frequency of walking than lower-educated mothers (*Mdn* = 1, *M* = 1.22). We found a shorter weekly duration of walking (*F*(1227) = 8.34, *η*^2^ = 0.01, *p* = 0.004) in employed mothers (*M_minutes_* = 184.18, *SD* = 142.10) as compared to mothers on maternity leave (*M_minutes_* = 216.64, *SD* = 165.89) or mothers without a paid job (*M_minutes_* = 224.93, *SD* = 186.19). There was also a difference for employment status in the frequency of walking on weekdays (*χ*^2^(2, *N* = 1275) = 17.54, *η*^2^ = 0.01, *p* = 0.000). Employed mothers walked approximately ‘one to three times’ (*Mdn* = 1, *M* = 1.57), while mothers on maternity leave (*Mdn* = 2, *M* = 1.74) and mothers without a paid job (*Mdn* = 2, *M* = 1.86) walked approximately ‘four to six times’ on weekdays. The association of employment status with weekly walking duration as well as walking frequency on weekdays is illustrated in Figure 1.

There was a difference in the daily duration of carrying for employment status (*χ*^2^(2, *N* = 1173) = 20.90, *η*^2^ = 0.02, *p* = 0.000). Employed mothers (*Mdn* = 1, stands for ‘less than one hour daily’, *M* = 0.74) showed a slightly shorter daily duration of carrying than mothers on maternity leave (*Mdn* = 1, *M* = 0.98) and mothers without a paid job (*Mdn* = 1, *M* = 1.13). Higher-educated mothers (*Mdn* = 1, *M* = 0.88) carried the infant for a slightly longer daily duration (*U* = 125,063, *N* = 1173, *r* = 0.03, *p* = 0.015) compared to lower-educated mothers (*Mdn* = 1, *M* = 0.78). Higher-educated mothers (*Mdn* = 1, stands for ‘sometimes’, *M* = 0.96) also carried slightly more frequently during outdoor walks (*U* = 140,097, *N* = 1233, *r* = 0.07, *p* = 0.022) compared to lower-educated mothers (*Mdn* = 1, *M* = 0.85). A higher frequency of carrying during outdoor walks (*U* = 55,673, *N* = 1233, *r* = 0.00, *p* = 0.005) was reported by mothers with one or more mental health issues (*Mdn* = 1, *M* = 1.15) as compared to mothers without (*Mdn* = 1, *M* = 0.91). Mothers with one or more mental health issues (*Mdn* = 1, *M* = 1.03) also showed a slightly longer daily duration of carrying (*U* = 53,318, *N* = 1173, *r* = 0.06, *p* = 0.027) compared to mothers with no mental health issues (*Mdn* = 1, *M* = 0.83).

3. Environmental Characteristics

For mother–infant dyads with more different types of recreational areas nearby, we found a longer weekly duration of walking (*r*(1227) = 0.10, *p* = 0.000, 95%CI [0.05, 0.16]) and a higher frequency of walking on weekdays (*r*(1273) = 0.07, *p* = 0.020, 95%CI [0.01, 0.12]) and on weekends (*r*(1269) = 0.08, *p* = 0.007, 95%CI [0.02, 0.13]). There was a small difference in the weekly duration of walking for housing type (*F*(1222) = 5.18, *η*^2^ = 0.004, *p* = 0.023). Mother–infant dyads engaged in walking for 188 min (*SD* = 162) living in detached houses, 180 min (*SD* = 133) in semidetached houses, 201 min (*SD* = 158) in terraced houses and 221 min (*SD* = 164) in apartments. We also found a difference for housing type in the frequency of walking on weekdays (*χ*^2^(4, *N* = 1275) = 16.45, *η*^2^ = 0.01, *p* = 0.002). Mother–infant dyads living in detached houses walked approximately ‘one to three times’ (*Mdn* = 1, *M* = 1.41), and those in semidetached (*Mdn* = 2, *M* = 1.66) and terraced houses (*Mdn* = 2, *M* = 1.66) as well as apartments (*Mdn* = 2, *M* = 1.78) walked approximately ‘four to six times’. A shorter weekly duration of walking (*t*(1227) = 3.02, *d* = 0.19, *p* = 0.003, 95%CI [0.35, 1.67]) was found when there was more than one child in the household (*M_minutes_* = 181.69, *SD* = 152.88) as compared to only one (*M_minutes_* = 205.11, *SD* = 155.18). The frequency of walking was also lower on weekdays (*U* = 188,305, *N* = 1275, *r* = 0.07, *p* = 0.017) when there was more than one child in the household (*Mdn* = 1, *M* = 1.56) as compared to only one (*Mdn* = 2, *M* = 1.68).

There was a small seasonal difference in the weekly duration of walking (*F*(1225) = 4.20, *η*^2^ = 0.01, *p* = 006), with 171.97 min (*SD* = 138.21) in the winter, 197.20 (*SD* = 153.61) in spring, 211.03 (*SD* = 159.81) in summer and 206.54 (*SD* = 163.54) in fall. There was also a difference for the frequency of walking on weekdays (*χ*^2^(3, 1275) = 12.21, *η*^2^ = 0.01, *p* = 0.007), with ‘one to three’ in the winter (*Mdn* = 1, *M* = 1.53) and ‘four to six’ in spring (*Mdn* = 2, *M* = 1.63), fall (*Mdn =* 2, *M* = 1.67) and summer (*Mdn =* 2, *M* = 1.73). Likewise, there was a difference in the frequency of walking on weekends (*χ*^2^(3, 1271) = 10.31, *η*^2^ = 0.01, *p* = 0.016), with the lowest frequency in the winter (*Mdn* = 1, *M* = 1.07), followed by spring (*Mdn* = 1, *M* = 1.12), fall (*Mdn =* 1, *M* = 1.16) and summer (*Mdn =* 1, *M* = 1.16). 

A longer daily duration of carrying (*r*(1171) = 0.08, *p* = 0.009, 95%CI [0.02, 0.13]) and a higher frequency of using the carrier outdoors (*r*(1231) = 0.06, *p* = 0.028, 95%CI [0.01, 0.12]) were associated with more types of recreational areas nearby. More daily hours of carrying were found (*U* = 135,642, *N* = 1173, *r* = 0.07, *p* = 0.016) when there was more than one child in the household (*Mdn* = 1, *M* = 0.96) as compared to one child (*Mdn* = 1, *M* = 0.80), and the carrier was used more frequently on walks (*U* = 149,088, *N* = 1232, *r* = 0.07, *p* = 0.009) when there was more than one child in the household (*Mdn* = 1, *M* = 1.05) as compared to one child (*Mdn* = 1, *M* = 0.88). The carrier was also used more frequently on outdoor walks (*U* = 23,569, *N* = 1233, *r* = 0.07, *p* = 0.010) when there was more than one adult in the household (*Mdn* = 1, *M* = 0.94) as compared to having no other adult in the household (*Mdn* = 0, stands for ‘(almost) never’, *M* = 0.68).

4. Boruta Results for Outdoor Walking and Carrying

**Figure 2:**
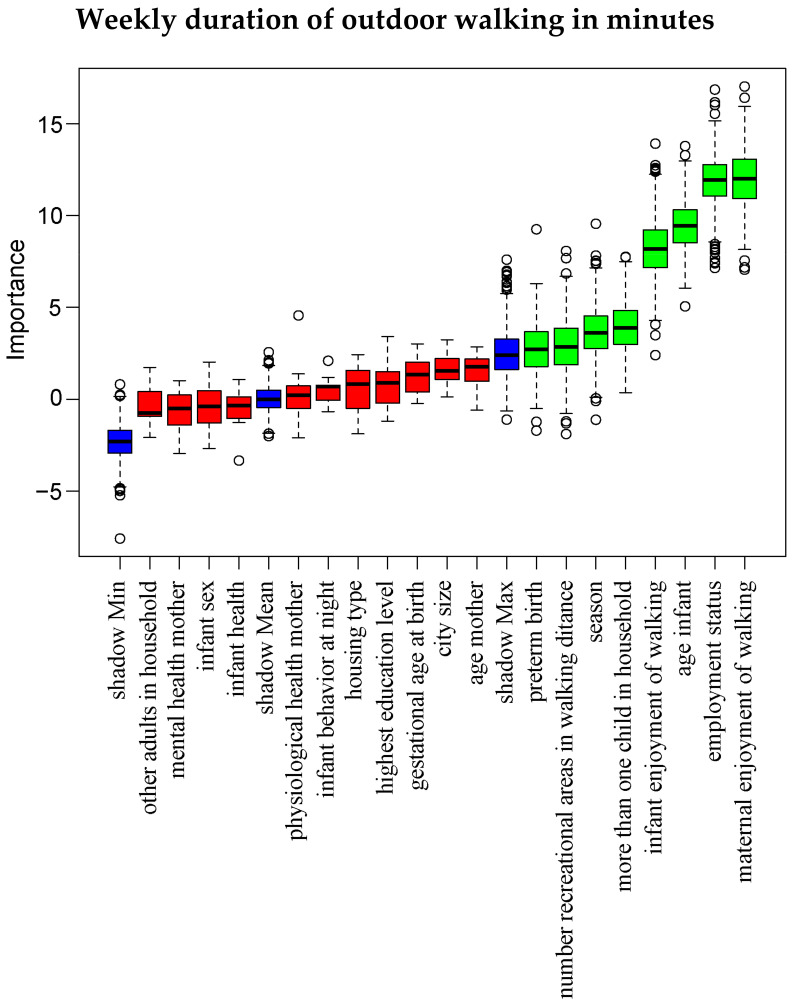
Boruta analysis predicting outdoor walking. Wrapped around the random forest algorithm, Boruta tests the importance of each variable against that of shadow variables created through shuffling the original variables. Green variables are classified as important, whereas red variables are unimportant. Blue variables show minimal, medium and maximal importance of the shadow variables.

Figure 2 displays Boruta results for the weekly duration of walking. In descending importance, a longer duration was predicted by: greater maternal enjoyment of walking, employment status (most in mothers without a paid job), younger infants, greater infant enjoyment of being walked, only one child in the household, season (lowest in winter), more recreational areas in walking distance and preterm birth.

**Figure 3:**
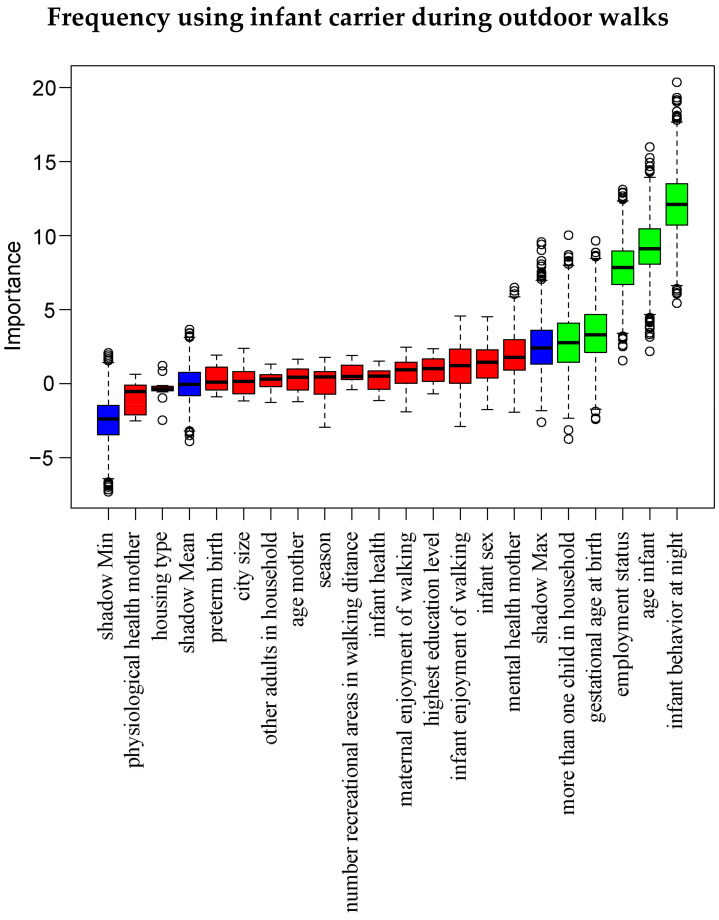
Boruta analysis predicting frequency of using infant carrier during outdoor walks. Wrapped around the random forest algorithm, Boruta tests the importance of each variable against that of shadow variables created through shuffling the original variables. Green variables are classified as important, whereas red variables are unimportant. Blue variables show minimal, medium and maximal importance of the shadow variables.

Figure 3 shows Boruta results for the frequency of using the infant carrier during outdoor walks. A higher frequency was predicted by: higher scores on infant behavior at night, younger infants, employment status (least for employed mothers), higher gestational age at birth and more than one child in the household.

##### Infant Outdoor Sleeping

All results of the analyses on the associations between sample characteristics with infant outdoor sleeping are presented in Table 3. All significant findings on infant outdoor sleeping after correcting for multiple testing are summarized in the following sections.

1. Infant Characteristics

The younger the infants, the more likely they were to be placed outdoors to sleep (*Z*(1164) = 3.65, *odds ratio (OR)* = 0.02, *p* = 0.000, 95%CI [0.01, 0.03]). Younger infant age was also associated with longer weekly durations (*r*(317) = −0.14, *p* = 0.015, 95%CI [−0.25, −0.03]) and a higher weekly frequency of outdoor sleeping (*r*(340) = −0.13, *p* = 0.013, 95%CI [−0.24, −0.03]).

2. Maternal Characteristics

Infants of higher-educated mothers (32.51%) were more likely to be placed outdoors to sleep (*χ*^2^(1, *N* = 1166) = 13.84, *Cohen’s ω* = 0.11, *p* = 0.000) compared to infants of lower-educated mothers (21.11%). Infants of employed mothers (34.89%) were also more likely to be placed outdoors to sleep (*χ*^2^(2, *N* = 1166) = 26.91, *Cohen’s ω* = 0.15, *p* = 0.000) than those of mothers on maternity leave (22.81%) or mothers without a paid job (17.50%).

3. Environmental Characteristics

The smaller the city, the more likely it was that infants were placed outdoors to sleep (*Z*(1164) = −2.33, *OR* = −0.13, *p* = 0.020, 95%CI [−0.24, −0.02]) and the longer the weekly duration of outdoor sleeping (*r*(317) = −0.16, *p* = 0.004, 95%CI [−0.27, −0.05]). The more types of recreational areas in walking distance, the more likely it was that infants were placed outdoors to sleep (*Z*(1164) = 2.77, *OR* = 0.18, *p* = 0.006, 95%CI [0.05, 0.31]). There was a difference for housing type (*χ*^2^(3, *N* = 1166) = 37.82, *Cohen’s ω* = 0.18, *p* = 0.000), with 45.21% of mothers in detached houses, 34.50% in semi-detached houses, 27.52% in terraced houses and 14.91% in apartments placing their infants outdoors to sleep. Lastly, there was a seasonal difference for the likelihood of infants being placed outdoors to sleep (*χ*^2^(3, *N* = 1166) = 15.98, *Cohen’s ω* = 0.12, *p* = 0.001), with 36.07% in the summer, 30.22% in the fall, 27.17% in the spring and 21.94% in the winter.

4. Boruta Analyses for Infant Outdoor Sleeping

**Figure 4:**
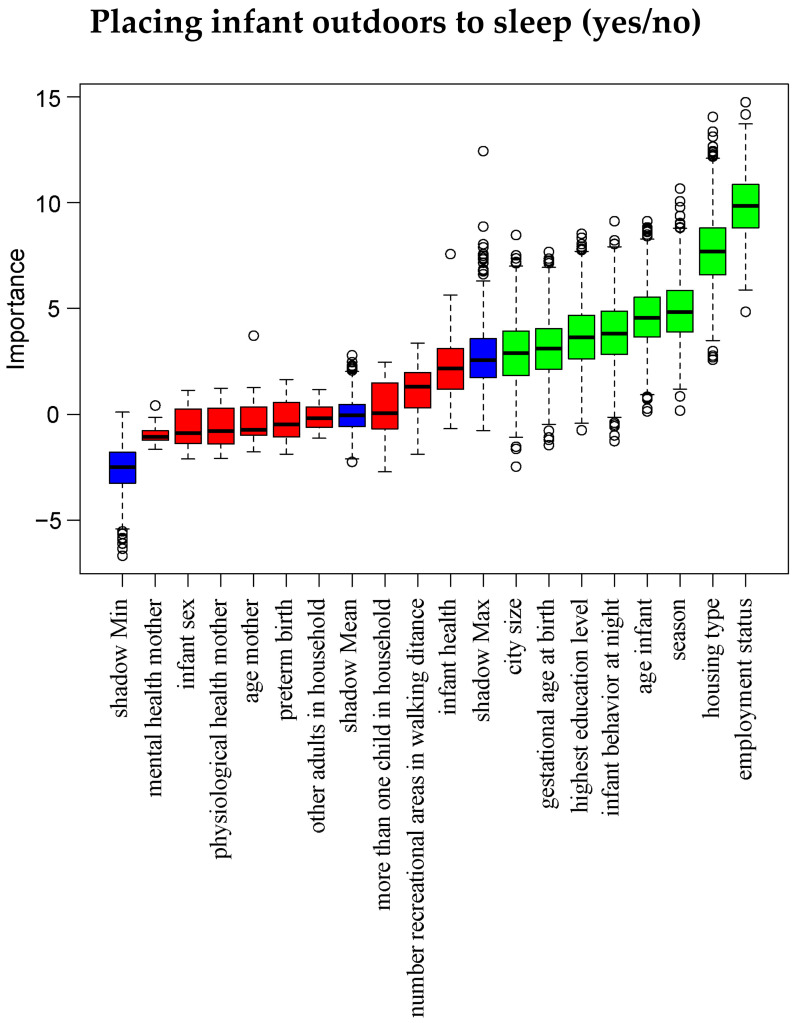
Boruta analysis predicting whether the infant is placed outdoors to sleep. Wrapped around the random forest algorithm, Boruta tests the importance of each variable against that of shadow variables created through shuffling the original variables. Green variables are classified as important, whereas red variables are unimportant. Blue variables show minimal, medium and maximal importance of the shadow variables.

Figure 4 displays Boruta results for infant outdoor sleeping. Predictive of placing the infant outdoors to sleep were: employment status (highest in employed mothers), housing type (least in apartments), season (least in the winter), younger infants, higher scores on infant behavior at night, higher maternal education, lower gestational age at birth and smaller city size.

## 4. Discussion

The first aim of the current study was to quantify how much time infants in the first year of life spend outdoors: being walked and carried and sleeping outdoors. Secondly, we investigated which infant, maternal and environmental factors were associated with time spent outdoors.

### 4.1. Outdoor Walking

We found that mothers walked approximately 201 min weekly with their infants, and most walked one to three times on weekdays and an additional one to three times on the weekend. Half of the mothers indicated that they would like to walk more. The most commonly reported reasons for walking were to reach a destination (84% of mothers) and leisure (82% of mothers), whereas the most common reasons for not walking were the weather (79% of mothers) and a lack of time (44% of mothers).

Statistical analyses indicated that only a few infant characteristics were predictive of outdoor walking. During weekdays, younger infants were taken on walks more frequently. This might be explained by the fact that mothers are usually on maternity leave when the infant is younger, and thus the mother may have more time for walking during weekdays. We also found a longer weekly duration of outdoor walking in preterm infants, and lower gestational age at birth was associated with more walks on weekdays. Note that mothers in the Netherlands are entitled to 16 weeks of maternity leave, which can be divided between the pre- and postnatal phase. When mothers give birth before the due date, they usually have more weeks of maternity leave after birth and possibly more time to go outdoors with their infants in this period. Furthermore, infants who enjoyed outdoor walks more were taken on walks for a longer weekly duration and more frequently during weekdays, as well as during the weekend.

Likewise, greater maternal enjoyment was an important predictor for longer durations of walking and a higher frequency of walks on weekdays and on weekends. Another predictor of outdoor walking was maternal employment status. Employed mothers reported a shorter weekly duration of walking and a lower frequency of walks, particularly during weekdays, as compared to mothers on maternity leave or mothers without a paid job, possibly due to having less time. Notably, research suggests that extended maternity leave is associated with improved infant and maternal health outcomes [66]. Current findings might indicate that mothers on maternity leave may have more time for recuperative activities, such as outdoor walking with their infants, which, in turn, might improve maternal and infant health. The current findings are in accordance with previous studies on toddlers and preschoolers, where less outdoor play was reported by employed mothers [15,42,44]. In the past decades, female education and employment rates have increased, and parents have come to rely more on childcare centers [67,68,69]. This trend might have led to decreased time outdoors for mother–infant dyads. How much time infants actually spend outdoors in childcare needs to be investigated in future studies in order to obtain a more comprehensive view of infants’ total outdoor time.

Mothers with a lower education walked more frequently with their infants on weekdays as well as on weekends. This is in accordance with studies in older children reporting more outdoor time for children of parents of lower education [15,43,44,45]. The current findings might be explained by the fact that higher education increases the likelihood of workforce engagement in women [70], leaving less time for outdoor walks. Indeed, post hoc analyses in our study showed that higher-educated mothers were more likely to be employed (*χ*^2^(1, *N* = 1275) = 77.88, *Cohen’s ω* = 0.25, *p* = 0.000) and worked for more hours weekly (*U* = 37,024, *N =* 774, *r* = 0.25, *p* = 0.000).

When there were more types of recreational areas within walking distance, mother–infant dyads engaged in outdoor walking for a longer duration weekly and more frequently on weekdays as well as on weekends. Likewise, previous studies reported more outdoor time in older children who lived in rural areas and areas with more greenery in the environment [45,46,47,48]. Additionally, dyads living in apartments engaged in outdoor walking for a longer duration weekly and more frequently on weekdays than dyads living in detached houses. Future research is of interest, assessing underlying reasons for differences depending on housing type. For instance, we did not assess whether families living in detached houses might spend more time outdoors around their house (e.g., in their yard). While more outdoor walking by dyads living in apartments might be explained by mothers compensating for restricted indoor space, as well as a lack of a yard, living in apartments might also be associated with lower education, which, in turn, was also related to more outdoor walking in the current study. Also, living in an apartment may be associated with shorter distances to shops, schools, health facilities, etc., allowing mothers to walk with their infants instead of taking the car or public transport. Having more than one child in the household was associated with a shorter weekly duration of walking and a shorter frequency of walking on weekdays, which may potentially be due to a lack of time because of increased caregiving responsibilities. Lastly, in accordance with previous studies, longer durations of outdoor walking and more frequent walks on weekdays and on weekends were found in warmer seasons [12,49].

### 4.2. Outdoor Carrying

In total, 22% of mothers reported using an infant carrier for half of the time or more during outdoor walks. Infants scoring higher on infant behavior at night (how much attention the infant needs at night, how much difficulty the infant has falling asleep and how often the infant wakes up at night) were carried more often during outdoor walks. One potential explanation for this finding is that these infants have more challenges sleeping at night and that the mothers use outdoor carrying as a way of facilitating (daytime) sleep through physical contact and movement [36,71]. On the other hand, given the non-causal nature of the findings, the possibility that being carried outdoors more leads to changes in infant behavior at night is just as likely, for instance, through carrying facilitating more sleep during the day and hence leading to less sleep at night. In addition, a third, non-measured variable may be explaining both outdoor carrying and behavior at night. For instance, breastfeeding may lead to more waking at night to feed as well as to mothers carrying their infant more often. Additionally, most typically, developing infants wake up regularly at night (e.g., signaling the need for being fed), and need help resettling to sleep, without this being considered problematic sleeping behavior [72,73,74]. Hence, future research is needed to disentangle the potential mechanisms underlying this finding.

Higher-educated mothers used the infant carrier more frequently for outdoor walks. Mothers having a mental health issue reported using the carrier slightly more frequently during outdoor walks than mothers without mental health issues. Again, the current study cannot assess the directionality of this association nor rule out other underlying factors not assessed in this survey. Future research assessing the association of both outdoor carrying and maternal mental health issues with infant sleeping behavior would be especially interesting to further understand underlying mechanisms. The carrier was also used more frequently during outdoor walks when there was another adult living in the household, if there were more types of recreational areas nearby and if mothers had more than one child. These factors could be related to practical reasons for using the carrier (e.g., having free hands when walking with more children), but future hypothesis-driven research is needed to investigate this.

### 4.3. Outdoor Sleeping

Outdoor sleeping was practiced with 29% of infants for approximately four hours a week and with a frequency of one to two times weekly. Outdoor sleeping was more likely in younger infants, and younger infants were placed outdoors more frequently and for a longer weekly duration, which might be explained by younger infants taking more naps in general [75]. Additionally, older infants are more mobile, and hence outdoor sleeping might be perceived as less practical or safe by the caregivers. In contrast, a study in Scandinavia, where outdoor sleeping is more popular, reported that most infants sleep outdoors throughout the first year of life [39].

We found a higher likelihood of outdoor sleeping in infants of mothers with a higher level of education and in working mothers. Notably, outdoor sleeping is often practiced in Dutch childcare centers, which might explain why infants of working mothers were placed outdoors to sleep more in the current sample. Infants living in areas with more types of recreational areas nearby and infants living in apartments or terraced houses were less likely to be placed outdoors to sleep than infants living in detached houses. Accordingly, parents in Scandinavia report cigarette smoke from neighboring balconies as a concern during infant outdoor sleeping [39]. We also found that infants in larger cities were less likely to be placed outdoors to sleep and slept outdoors for fewer hours weekly. Living in a larger city might lead to less private outdoor space, as well as increased air pollution and more parental safety concerns, but these potential explanations need to be examined in future studies. Nonetheless, these findings are in accordance with studies on outdoor play in older children, where more outdoor time was reported in rural areas [45,46,47,48].

Lastly, infants of mothers participating in the winter were placed outdoors to sleep less often. In contrast with these findings, a survey in Scandinavian parents found −6 degrees Celsius to be the most preferred temperature for infant outdoor sleeping [38,39]. The authors suggest that colder outdoor temperatures allow for more swaddling through additional layers of clothing, which restricts infants’ movement and potentially increases sleep duration, as longer sleep durations were reported outdoors compared to indoors [38]. In the Netherlands, average temperatures range from +17 degrees Celsius in the summer to +3 in the winter, suggesting that outdoor sleeping is possible also in the winter.

### 4.4. Limitations, Strengths and Spin-Off Questions

The current study has some limitations. The observational and cross-sectional nature of the study design, as well as the non-standardized survey, restrict interpretability and preclude us from drawing conclusions on the causality of associations. In addition, we solely focused on outdoor walking and carrying performed by mothers and relied on maternal report. Also, the variable infant behavior at night was assessed through a single item collapsing all three nightly behaviors and we did not ask parents whether they perceived the infants’ nightly sleeping behavior as problematic. Factors that could interact with outdoor activities (e.g., partner support, culture, perception and safety of the outdoors) were not examined in this study and may be important explanatory variables to include in future research. In addition, future studies should consider collecting more objective measures of outdoor time through the use of wearables and apps designed to register walks. Furthermore, future work should also assess outdoor time with other caregivers, such as fathers and grandparents, as well as outdoor time in childcare centers. The homogeneous nature of the sample (i.e., 73% higher education and 95% Dutch) restricts generalizability of our findings to other groups. Lastly, the current study did not assess all types of activities commonly performed with infants outdoors, such as awake time in the yard or biking with the infant, and hence does not provide an insight in the total amount of time infants spend outdoors.

Nevertheless, the current study has several strengths. This is one of the first studies in this relevant area of research and the large sample size allowed for a data-driven approach. Also, the Boruta algorithm used is a powerful tool to reveal the importance of variables, providing a comprehensive insight into demographic characteristics associated with outdoor walking, carrying and outdoor sleeping during infancy. The exploratory, data-driven approach of the current study can deliver important insights for future hypothesis-driven research. Furthermore, the study delivers crucial input for future research on interventions to facilitate outdoor activities with infants. For instance, more than half of the mothers in this study reported walking in order to benefit their own health, supporting the idea that outdoor walking might also be of interest for interventions targeted at improving maternal postnatal health [41]. Furthermore, maternal enjoyment of outdoor walks was one of the strongest predictors of outdoor walking with the infant. This implies that future interventions with the aim to facilitate postnatal outdoor walking may target maternal enjoyment of the activity, for instance, by facilitating group walks or making mothers aware of recreational areas suitable for enjoyable walks. Finally, the current findings on reduced outdoor walking in employed mothers during weekdays might prompt future research into the potential benefits of longer maternity leaves for mother and child.

### 4.5. Conclusions

This study identified associations between infant, maternal and environmental characteristics and infant time spent being walked or sleeping outdoors in the first year of life. Summarizing, more mother–infant outdoor walking was related to younger infant age, mothers without a paid job or on maternity leave and more recreational areas nearby. More outdoor sleeping was associated with younger infant age, higher maternal education and living in detached houses and smaller cities. These results lay a solid foundation for future hypothesis-driven research on the effects of the outdoors on child development as well as on facilitators and barriers for caregivers. Future studies should include other caregivers besides the mothers and assess cultural differences as well as parental perceptions of the outdoors. Ultimately, this line of work can inform advice for parents, governmental policies and urban planning related to bringing up healthy future generations.

## Figures and Tables

**Table 1:** Descriptive statistics.

Outdoor Walking and Carrying	M (*SD*; Range) or *N* (%)	Mis.
Weekly duration of outdoor walking in minutes ^a^	201.28 (*SD* = 170; 0–1600)	46
Frequency of outdoor walking on weekdays (Monday to Friday) ^b^		n/a
(Almost) never	69 (5.41%)	
1–3	548 (42.98%)	
4–6	472 (37.02%)	
7–9	137 (10.75%)	
≥10	49 (3.84%)	
Frequency of outdoor walking on weekends (Saturday to Sunday) ^b^		4
(Almost) never	60 (4.72%)	
1–3	1019 (80.17%)	
4–6	163 (12.82%)	
7–9	21 (1.65%)	
≥10	8 (0.63%)	
Frequency of carrying the infant during outdoor walks ^b^		42
(Almost) never	498 (40.39%)	
Sometimes	464 (37.63%)	
Half of the time	157 (12.73%)	
Most of the time	84 (6.81%)	
Always	30 (2.43%)	
Daily duration of infant carrying in hours (indoors + outdoors) ^b^		102
(Almost) never	468 (39.90%)	
<1 h	496 (42.28%)	
1–2 h	147 (12.53%)	
3–4 h	45 (3.84%)	
5–6 h	14 (1.19%)	
≥7 h	3 (0.26%)	
Satisfaction with the amount of walking ^c,e^		71
Satisfied	602 (50.00%)	
Would like to walk more	598 (49.67%)	
Would like to walk less	4 (0.33%)	
Subjective reasons for walking ^d,e^		48
Reaching a destination	1026 (83.62%)	
Leisure	1012 (82.48%)	
Maternal health	712 (58.03%)	
Facilitating infant sleep/soothing	337 (27.47%)	
Walking a dog	206 (16.79%)	
Subjective reasons against walking ^d,e^		72
Weather	951 (79.05%)	
Lack of time (e.g., due to work)	535 (44.47%)	
Easier to go by car	280 (23.28%)	
Maternal health issues	258 (21.45%)	
Not feeling like it	248 (20.62%)	
Infant health issues	132 (10.97%)	
No good walking environment	127 (10.56%)	
Too much traffic	30 (2.49%)	
**Infant outdoor sleeping**		
Placing infant outdoors to sleep (yes) ^c^	343 (29.42%)	109
Weekly duration of outdoor sleeping in hours ^a,f^	4.31 (*SD* = 5.27; 0–39)	24
Weekly frequency of outdoor sleeping ^b,f^		1
<1	117 (34.21%)	
1–2	148 (43.27%)	
3–4	55 (16.08%)	
5–6	14 (4.09%)	
≥7	8 (2.34%)	
**Infant characteristics**		
Age in weeks ^a^	23.57 (*SD =* 13.87; 0–52)	n/a
Sex (girl) ^c^	630 (49.41%)	n/a
Gestational age at birth in week s ^a^	39.27 (*SD =* 1.79; 28–42)	2
Preterm (<37 weeks) ^c^	77 (6.05%)	2
One or more health issues (yes) ^c,g^	105 (8.24%)	n/a
Infant behavior at night ^b^		94
Almost never needs attention/falls asleep easily/almost never wakes up	341 (26.75%)	
Needs attention very occasionally/wakes very occasionally	396 (33.53%)	
Needs regular attention/sometimes wakes	334 (28.28%)	
Needs a lot of attention/has difficulty falling asleep/wakes up often	110 (9.31%)	
Infant’s enjoyment of being walked ^a^	83.85 (*SD* = 15.95; 1–100)	66
**Maternal characteristics**		
Age in years ^a^	31.44 (*SD =* 4.31; 18–50)	n/a
One or more mental health issues (yes) ^c,g^	120 (9.41%)	n/a
One or more physical health issues (yes) ^c,g^	229 (17.96%)	n/a
Education level ^c^		n/a
Lower	356 (27.92%)	
Higher	919 (72.08%)	
Employment status ^c^	n/a	
Working	774 (60.71%)	
Maternity leave	370 (29.02%)	
No paid job	131 (10.27%)	
Weekly working hours (in employed mothers, *N* = 774) ^b^		n/a
0–8	19 (2.45%)	
9–16	28 (3.62%)	
17–24	184 (23.77%)	
25–32	368 (47.55%)	
33–40	168 (21.71%)	
>40	7 (0.90%)	
Maternal enjoyment of walking with infant ^a^	84.60 (*SD* = 15.42; 1–100)	63
**Environmental characteristics**		
More than one child in household (yes) ^c^	399 (31.29%)	n/a
More than one adult in household (yes) ^c^	1221 (95.76%)	n/a
City size (number of citizens) ^b^		n/a
≤5000	395 (30.98%)	
≤20.000	279 (21.88%)	
≤100.000	293 (22.98%)	
>100.000	308 (24.16%)	
Types of recreational areas nearby ^d,e^		n/a
City park	625 (49.02%)	
Green square	631 (49.49%)	
Forest	595 (46.67%)	
National park or nature reserve	88 (6.90%)	
Other	100 (7.84%)	
None	32 (2.51%)	
Sum of types of recreational areas nearby ^a^	1.60 (*SD =* 0.98; 0–4)	n/a
House ^c^		n/a
Detached	157 (12.31%)	
Semidetached	256 (20.08%)	
Terraced	685 (53.73%)	
Apartment	172 (13.49%)	
Other	5 (0.39%)	
Season during participation ^c^		n/a
Spring (March–May)	298 (23.37%)	
Summer (June–August)	278 (21.80%)	
Fall (September–November)	399 (31.29%)	
Winter (December–February)	300 (23.53%)	

Note. n/a = no missing data. Mis. = Missing. M = mean. *SD* = standard deviation. ^a^ Continuous, ^b^ Ordinal, ^c^ Categorical, ^d^ List, ^e^ Not used in further analyses, ^f^ Includes only infants who were reported to sleep outdoors in the previous item (*N* = 343), ^g^ If ‘yes’ was selected, mothers were asked to provide a description of the health issues (not used in further analyses).

**Table 2:** Associations of outdoor walking and carrying with sample characteristics.

	Weekly Duration of Walking in Minutes ^g^		Frequency Walking Weekdays		Frequency Walking Weekends		Frequency Carrying Outdoors		Daily Carrying Hours (Indoors + Outdoors)	
**Infant**	Statistic (df)	*p*	Statistic (df)	*p*	Statistic (df)	*p*	Statistic (df)	*p*	Statistic (df)	*p*
Age	−0.04 (1227) ^a^	0.168	−0.08 (1273) ^b^	0.004 *	−0.03 (1269) ^b^	0.308	−0.03 (1231) ^b^	0.235	−0.14 (1171) ^b^	0.000 *
Sex (boy/girl)	−0.76 (1227) ^c^	0.447	198,899 ^e^	0.485	194,613 ^e^	0.109	189,372 ^e^	0.916	166,958 ^e^	0.358
Gestational age at birth	−0.04 (1225) ^a^	0.135	−0.07 (1271) ^b^	0.018 *	−0.01 (1267) ^b^	0.647	0.05 (1229) ^b^	0.061	0.02 (1169) ^b^	0.548
Preterm (yes/no)	−2.30 (1225) ^c^	0.022 *	40,382 ^e^	0.052	45,078 ^e^	0.707	44,438 ^e^	0.697	39,236 ^e^	0.942
Health issues (yes/no)	1.43 (1227) ^c^	0.152	64,364 ^e^	0.383	63,948 ^e^	0.275	59,138 ^e^	0.652	52,345 ^e^	0.207
Infant behavior at night	−0.04 (1179) ^b^	0.182	−0.05 (1179) ^b^	0.067	−0.03 (1179) ^b^	0.336	0.16 (1179) ^b^	0.000 *	0.20 (1171) ^b^	0.000 *
Infant enjoyment of outdoor walks	0.22 (1207) ^b^	0.000 *	0.18 (1207) ^b^	0.000 *	0.22 (1207) ^b^	0.000 *	0.03 (1207) ^b^	0.337	0.03 (1171) ^b^	0.254
**Maternal**										
Age	−0.04 (1227) ^a^	0.179	−0.06 (1273) ^b^	0.033	−0.03 (1269) ^b^	0.237	0.03 (1231) ^b^	0.350	0.01 (1171) ^b^	0.667
Education level (higher/lower)	0.38 (1227) ^c^	0.707	188,846 ^e^	0.000 *	174,236 ^e^	0.004 *	140,097 ^e^	0.022 *	125,063 ^e^	0.015 *
Employment (employed/mat.leave/no paid job)	8.34 (1227) ^d^	0.004 *	17.54 (2) ^f^	0.000 *	1.21 (2) ^f^	0.546	5.42 (2) ^f^	0.067	20.90 (2) ^f^	0.000 *
Weekly working hours (in employed mothers)	−0.00 (744) ^b^	0.990	−0.06 (772) ^b^	0.076	0.02 (770) ^b^	0.572	0.02 (749) ^b^	0.558	−0.03 (710) ^b^	0.362
Mental health issues (yes/no)	−0.10 (1227) ^c^	0.921	63,676 ^e^	0.116	69,050 ^e^	0.997	55,673 ^e^	0.005 *	53,318 ^e^	0.027 *
Physiological health issues (yes/no)	1.18 (1227) ^c^	0.236	120,048 ^e^	0.952	0.124862 ^e^	0.112	119,016 ^e^	0.156	97,485 ^e^	0.186
Maternal enjoyment of walking	0.24 (1210) ^b^	0.000 *	0.20 (1210) ^b^	0.000 *	0.23 (1210) ^b^	0.000 *	0.03 (1210) ^b^	0.259	0.03 (1171) ^b^	0.359
**Environmental**										
City size	0.06 (1227) ^b^	0.037	−0.01 (1273) ^b^	0.851	0.01 (1269) ^b^	0.615	0.02 (1231) ^b^	0.451	0.04 (1171) ^b^	0.170
Types of different recreational areas nearby	0.10 (1227) ^b^	0.000 *	0.07 (1273) ^b^	0.020 *	0.08 (1269) ^b^	0.007 *	0.06 (1231) ^b^	0.028 *	0.08 (1171) ^b^	0.009 *
House (detached/semidetached/terraced/apartment)	5.18 (1222) ^d^	0.023 *	16.45 (4) ^f^	0.002 *	5.97 (3) ^f^	0.113	4.94 (3) ^f^	0.176	8.27 (3) ^f^	0.041
More than one child in household (yes/no)	3.02 (1227) ^c^	0.003 *	188,305 ^e^	0.017 *	180,393 ^e^	0.090	149,088 ^e^	0.009 *	135,642 ^e^	0.016 *
More than one adult in household (yes/no)	−0.35 (1227) ^c^	0.725	33,425 ^e^	0.662	31,974 ^e^	0.865	23,569 ^e^	0.010 *	24,995 ^e^	0.494
Season (spring/summer/fall/winter)	4.20 (1225) ^d^	0.006 *	12.21 (3) ^f^	0.007 *	10.31 (3) ^f^	0.016 *	2.75 (3) ^f^	0.431	0.90 (3) ^f^	0.826

Note. df = degrees of freedom. Mat.leave = Maternity leave. * *p*-values printed in bold were significant after the Benjamini–Hochberg correction. ^a^ Pearson correlation coefficient for continuous data, ^b^ Spearman’s rho for ordinal or non-normally distributed residuals, ^c^ *T*-statistic for independent samples *t*-tests comparing two groups, ^d^ Cohen’s *F* for analyses of variance comparing more than two groups, ^e^ *U* for Mann–Whitney U tests comparing two groups with non-normally distributed residuals, ^f^ *χ*^2^ for Kruskal–Wallis test comparing more than two groups with non-normally distributed residuals, ^g^ Square-root-transformed and winsorized data.

**Table 3:** Associations of infant outdoor sleeping with sample characteristics.

	Placing Infant Outdoors to Sleep		Weekly Duration Outdoor Sleeping ^f^		Weekly Frequency Outdoor Sleeping	
**Infant**	Statistic (df)	*p*	Statistic (df)	*p*	Statistic (df)	*p*
Age	3.65 (1164) ^e^	0.000 *	−0.14 (317) ^a^	0.015 *	−0.13 (340) ^a^	0.013 *
Sex (boy/girl)	0.17 (1) ^d^	0.683	13,795 ^b^	0.181	15,032 ^b^	0.604
Gestational age at birth	1.04 (1162) ^e^	0.298	−0.10 (315) ^a^	0.061	−0.06 (340) ^a^	0.246
Preterm (yes/no)	0.60 (1) ^d^	0.437	2933 ^b^	0.355	3721.5 ^b^	0.830
Health issues (yes/no)	1.09 (1) ^d^	0.296	3892.5 ^b^	0.506	4949 ^b^	0.794
Infant behavior at night	0.75 (1163) ^e^	0.453	−0.05 (317) ^a^	0.382	−0.05 (339) ^a^	0.320
**Maternal**	Statistic (df)	*p*	Statistic (df)	*p*	Statistic (df)	*p*
Age	2.16 (1164) ^e^	0.031	0.04 (317) ^a^	0.479	0.04 (340) ^a^	0.408
Education level (higher/lower)	13.84 (1) ^d^	0.000 *	8323.5 ^b^	0.479	9582 ^b^	0.483
Employment (working/maternity leave/no paid job)	26.91 (2) ^d^	0.000 *	3.85 (2) ^c^	0.146	5.93 (2) ^c^	0.052
Weekly working hours (in employed mothers)	0.53 (706) ^e^	0.596	0.12 (231) ^a^	0.061	0.09 (245) ^a^	0.170
Mental health issues (yes/no)	0.00 (1) ^d^	0.999	4751 ^b^	0.746	4910 ^b^	0.710
Physiological health issues (yes/no)	0.03 (1) ^d^	0.863	6966 ^b^	0.634	8723 ^b^	0.949
**Environmental**	Statistic (df)	*p*	Statistic (df)	*p*	Statistic (df)	*p*
City size	−2.33 (1164) ^e^	0.020 *	−0.16 (317) ^a^	0.004 *	−0.08 (340) ^a^	0.118
Types of different recreational areas nearby	2.77 (1165) ^e^	0.006 *	0.06 (317) ^a^	0.254	0.08 (340) ^a^	0.132
House (detached/semidetached/terraced/apartment)	37.82 (3) ^d^	0.000 *	9.96 (4) ^c^	0.041	7.05 (04) ^c^	0.133
More than one child in household (yes/no)	3.47 (1) ^d^	0.063	11,331 ^b^	0.738	12,196 ^b^	0.151
More than one adult in household (yes/no)	1.97 (1) ^d^	0.160	1928.5 ^b^	0.046	1980.5 ^b^	0.075
Season (spring/summer/fall/winter)	15.98 (3) ^d^	0.001 *	8.10 (3) ^c^	0.044	3.12 (3) ^c^	0.373

* *p*-values printed in bold were significant after the Benjamini–Hochberg correction. ^a^ Spearman’s rho for ordinal data, ^b^ U for Mann–Whitney U tests comparing two groups with non-normally distributed residuals, ^c^ *χ*^2^ for Kruskal–Wallis test comparing more than two groups with non-normally distributed residuals, ^d^ *χ*^2^ for chi-square tests on categorical data, ^e^ Wald Z for binomial logistic regressions for continuous predictors and categorical outcome data, ^f^ Winsorized data.

## Data Availability

The first author has full access to the study data and takes responsibility for integrity and accuracy. The research data can be requested from the first author via e-mail due to privacy restrictions.

## Supplementary Materials

The following supporting information can be downloaded at: https://www.mdpi.com/article/10.3390/ijerph21060694/s1, Supplementary S1: Survey on Outdoor Activities of Mothers with Infants; Supplementary S2: Benjamini-Hochberg Corrections.
