# Supplementary material for: Infant Care: Predictors of Outdoor Walking, Infant Carrying and Infant Outdoor Sleeping
Source: Int J Environ Res Public Health. 2024 May 28;21(6):694. doi: 10.3390/ijerph21060694 (PMC11203610; doi:10.3390/ijerph21060694)
Supplement: Supplementary file 1 [file ijerph-21-00694-s001.zip › ijerph-2958631-supplementary.pdf]

Supplementary S1: Survey on Outdoor Activities of Mothers with Infants

Part 1: Demographic data: The questionnaire starts with a number of questions about yourself and your living environment.

1.1. How old are you in years?

1.2. Where were you born?

- o Netherlands
- o Other, namely:

1.3. What is your ethnic background?

- o European
- o Turkish
- o Moroccan
- o Antillean
- o Surinamese
- o Indonesian
- o African
- o Asian
- o Latin American
- o Other, namely:

1.4. What is your highest completed level of education?

- o Primary education
- o VMBO
- o MBO
- o HAVO
- o VWO (atheneum/gymnasium)
- o HBO
- o University
- o Other, namely:

1.5. Do you currently have a paid job?

- o yes
- o no

1.5.1. Can you describe your profession below?

1.5.2. How many hours per week do you normally work?

- o 0-8 hours (0-20%)
- o 9-16 hours (21-40%)
- o 17-24 hours (41-60%)
- o 25-32 hours (61-80%)
- o 33-40 hours (81-100%)
- o More than 40 hours (>100%)

1.5.3. Are you currently on maternity leave?

- o yes
- o no

1.6. Do you currently have a permanent partner?

- o yes

- no

1.7. How many people does your household consist of (including your baby)?

. . . adults (older than 18)

. . . children (younger than 18)

1.7.1. What are the ages of the children in your household?

1.8. Do you live in a:

- village (up to 5000 inhabitants)
- small city (up to 20,000 inhabitants)
- medium-sized city (up to 100,000 inhabitants)
- large city (more than 100,000 inhabitants)

1.9. What type of home do you currently live in?

- detached house
- semi-detached house
- terraced house
- apartment
- other, namely:

1.9.1. [If Apartment] On which floor do you live?

- ground floor
- first floor
- second floor
- third floor
- fourth floor or higher

1.10. What types of green recreational areas are there within walking distance of your home? [Multiple options possible]

- city park
- square
- wooded or tree-rich environment
- green area with walking paths
- national park
- On the street, in the city/village
- none
- other, namely

Part 2: The following questions are about the health and well-being of you and your baby:

2.1. Do you have a physical illness/condition or complaints?

- yes, namely:
- no

2.2. Do you have mental and/or psychological complaints yourself?

- Yes, namely:
- no

2.3. What was the duration of your pregnancy in days?

The following questions are about habits regarding walking outside before and during your pregnancy. By walking outside we mean a walk outside the house of at least 15 consecutive minutes.

2.4. <u>Before your pregnancy,</u> how often did you walk outside on average per week?

- (almost) never
- 1 to 3 times a week
- 4 to 6 times a week
- 7 to 9 times a week
- 10 times a week or more

2.5. <u>During your pregnancy,</u> how often did you walk outside on average per week?

- (almost) never
- 1 to 3 times a week
- 4 to 6 times a week
- 7 to 9 times a week
- 10 times a week or more

2.6. What is the sex of your baby?

- boy
- girl

2.7. Does your baby have an illness/condition or health problems?

- yes, namely:
- no

Part 2.2: Activities and habits

The following questions are about habits related to walking outside with your baby. By walking outside we mean a walk outside the house for at least 15 consecutive minutes.

<u>During the week,…</u>

2.8. How often do you usually walk with your baby **<u>during the week (Monday to Friday)</u>**?

- (almost) never
- 1 to 3 times a week
- 4 to 6 times a week
- 7 to 9 times a week
- 10 times a week or more

2.9. How often do you usually walk outside with your baby **<u>on weekends (Saturday to Sunday)</u>**?

- (almost) never
- 1 to 3 times per weekend
- 4 to 6 times per weekend
- 7 to 9 times per weekend
- 10 times or more per weekend

2.10. **<u>In one week</u>**, how long do you usually walk outside with your baby (total in minutes over 7 days)?

2.10.1. How often do you use a baby carrier/sling to walk with your baby outside the home?

- (almost) never
- sometimes
- half the time
- usually
- always

2.11. For what reasons do you walk outside with your baby? (Multiple answers possible)

o as a leisure activity

o to reach a destination, such as the store or childcare

o to walk the dog

o to get the baby to sleep or soothe the baby

o for my physical health

o other, namely:

2.12. When you walk outside the house with your baby, how often do you walk with other people? (e.g. your partner, other children, friends)

o never

o sometimes

o half the time

o most of the time

o always

2.13. Are there other people who regularly walk outside with your baby **without you**? (multiple options possible)

o no
o father of baby
o baby's brother or sister
o baby's grandmother or grandfather
o babysitter or nursery employee
o other, namely:

2.14. How much do you enjoy walking outside?

o slider from not at all to very much

2.15. How much does your baby enjoy it when you walk him/her outside?

o slider from not at all to very much

2.16. How satisfied are you with the amount of time you walk outside with your baby?

o I am satisfied
o I would like to walk more with my baby
o I would like to walk less with my baby

2.17. What are reasons for you to decide not to walk outside with your baby? (Multiple answers possible)

o it is easier to go by car
o there is too much traffic on the street
o there is no nice environment to walk in
o weather conditions
o my own health problems
o my baby's health problems
o no time
o not feeling like it
o other, namely:

2.18. Where do you usually walk outside with your baby? [Multiple options possible]

o city park
o square
o wooded or tree-rich environment
o green area with walking paths
o national park

o on the street, in the city/village

o other, namely

The following questions are about habits in the daily lives of you and your baby.

2.19. How does your baby fall asleep? [Multiple options possible]

- o during feeding
- o when he/she is being rocked
- o when he/she is held on your lap
- o while walking with the stroller
- o when he/she is carried in the arms
- o if he/she is carried in a baby carrier/sling
- o in bed, with a parent nearby
- o in bed, without a parent nearby
- o otherwise, namely….

2.20. How does your baby behave at night?

- o The baby needs a lot of attention/has difficulty falling asleep/wakes up often during the night.
- o The baby needs regular attention/sometimes wakes up during the night.
- o The baby needs attention very occasionally/wakes up very occasionally during the night.
- o The baby almost never needs attention/falls asleep easily/almost never wakes up at night.

2.21. Do you ever use a baby carrier/sling for your baby (indoors or outdoors)?

- o no never
- o I have only used this once or twice
- o yes, regularly
- o yes, often or always

2.21.2. [If one of the last two options was chosen] How many hours per day do you use a baby carrier/sling on average?

less than one hour per day

- o (almost) Never
- o Less than 1 hour per day
- o 1 to 2 hours per day
- o 3 to 4 hours per day
- o 5 to 6 hours per day
- o 7 hours or more per day

The following questions are about bathing habits for your baby.

2.22. How often do you bathe your baby?

- o Less than once a week
- o 1 to 2 times a week
- o up to 4 times a week
- o up to 6 times a week
- o 7 or more times a week

2.23. Do you use a washcloth to wash your baby during the bath?

- (almost) never
- yes

2.24. How long does it take you to give your baby a bath (from starting to undress to fully dressed again)?

- Less than 5 minutes
- 5 to 10 minutes
- 11 to 15 minutes
- 16 to 20 minutes
- More than 20 minutes

2.25. What do you use to bathe your baby?

- bathtub for babies
- tummy tub
- sink or sink in the countertop
- other, namely:

2.26 Is your baby put outside in the garden or on the balcony/terrace to sleep during the day (for example in a stationary pram or cot, by you or other people, including the childcare center)?

o no
o yes

2.26.2. [If yes] How many times a week does your baby sleep outside in the garden or on the balcony/terrace?

- 1 to 2 times a week
- 3 to 4 times a week
- 5 to 6 times a week
- 7 or more times a week

2.26.3. In one week, how long does your baby usually sleep outside in the garden or on the balcony/terrace (total in hours over 7 days)?

2.27. Do you have any further comments regarding this survey?

Supplementary S2: Benjamini-Hochberg Corrections

Table S1. Benjamini-Hochberg Correction for Aim 2

Predictor variable	Outcome	P-value	Rank	Critical value
Maternal enjoyment of walking	Walking weekly in minutes	8.32E-17	1	0.000649
Maternal enjoyment of walking	Frequency during weekends	3.77E-16	2	0.001299
Infant enjoyment of outdoor walks	Frequency during weekends	1.49E-14	3	0.001948
Infant enjoyment of outdoor walks	Walking weekly in minutes	1.97E-14	4	0.002597
Maternal enjoyment of walking	Frequency during weekdays	2.93E-12	5	0.003247
Infant behavior at night	Infant carrying (indoors+outdoors)	1.54E-11	6	0.003896
Infant enjoyment of outdoor walks	Frequency during weekdays	1.04E-10	7	0.004545
House (detached/semidetached/terraced/apartment)	Sleeping outdoors (Yes/No)	3.09E-08	8	0.005195
Infant behavior at night	Frequency carrying outdoors	4.64E-08	9	0.005844
Employment (working/maternity leave/unemployed)	Sleeping outdoors (Yes/No)	1.43E-06	10	0.006494

Infant age	Infant carrying (indoors+outdoors)	1.94E-06	11	0.007143
Education level (higher/lower)	Frequency during weekdays	4.30E-06	12	0.007792
Employment (working/maternity leave/unemployed)	Infant carrying (indoors+outdoors)	2.90E-05	13	0.008442
Employment (working/maternity leave/unemployed)	Frequency during weekdays	0.000156	14	0.009091
Education level (higher/lower)	Sleeping outdoors (Yes/No)	0.000199	15	0.009740
Infant age	Sleeping outdoors (Yes/No)	0.000263	16	0.010390
Types of different recreational areas nearby	Walking weekly in minutes	0.000301	17	0.011039
Season (spring/summer/fall/winter)	Sleeping outdoors (Yes/No)	0.001147	18	0.011688
House (detached/semidetached/terraced/apartment)	Frequency during weekdays	0.002468	19	0.012338
More than one child in household	Walking weekly in minutes	0.002618	20	0.012987
Employment (working/maternity leave/unemployed)	Walking weekly in minutes	0.003941	21	0.013636
City size	Weekly hours outdoor sleeping	0.004238	22	0.014286
Education level (higher/lower)	Frequency during weekends	0.004295	23	0.014935
Infant age	Frequency during weekdays	0.004416	24	0.015584
Maternal mental health issues (yes/no)	Frequency carrying outdoors	0.005125	25	0.016234
Types of different recreational areas nearby	Sleeping outdoors (Yes/No)	0.005643	26	0.016883
Season (spring/summer/fall/winter)	Walking weekly in minutes	0.005726	27	0.017532
Season (spring/summer/fall/winter)	Frequency during weekdays	0.006699	28	0.018182
Types of different recreational areas nearby	Frequency during weekends	0.007103	29	0.018831
Types of different recreational areas nearby	Infant carrying (indoors+outdoors)	0.008937	30	0.019481
More than one child in household	Frequency carrying outdoors	0.009161	31	0.020130
More than one adult in household	Frequency carrying outdoors	0.009612	32	0.020779
Infant age	Frequency outdoor sleeping	0.013188	33	0.021429
Infant age	Weekly hours outdoor sleeping	0.014533	34	0.022078
Education level (higher/lower)	Infant carrying (indoors+outdoors)	0.014809	35	0.022727
More than one child in household	Infant carrying (indoors+outdoors)	0.015670	36	0.023377
Season (spring/summer/fall/winter)	Frequency during weekends	0.016142	37	0.024026
More than one child in household	Frequency during weekdays	0.017137	38	0.024675
Gestational age at birth	Frequency during weekdays	0.017597	39	0.025325
Types of different recreational areas nearby	Frequency during weekdays	0.019521	40	0.025974
City size	Sleeping outdoors (Yes/No)	0.019778	41	0.026623
Preterm (yes/no)	Walking weekly in minutes	0.021606	42	0.027273
Education level (higher/lower)	Frequency carrying outdoors	0.022184	43	0.027922

House (detached/semidetached/terraced/apartment)	Walking weekly in minutes	0.023036	44	0.028571
Maternal mental health issues (yes/no)	Infant carrying (indoors+outdoors)	0.026970	45	0.029221
Types of different recreational areas nearby	Frequency carrying outdoors	0.027732	46	0.029870
Maternal age	Sleeping outdoors (Yes/No)	0.030790	47	0.030519
Maternal age	Frequency during weekdays	0.033012	48	0.031169
City size	Walking weekly in minutes	0.036775	49	0.031818
House (detached/semidetached/terraced/apartment)	Infant carrying (indoors+outdoors)	0.040783	50	0.032468
House (detached/semidetached/terraced/apartment)	Weekly hours outdoor sleeping	0.041122	51	0.033117
Season (spring/summer/fall/winter)	Weekly hours outdoor sleeping	0.044046	52	0.033766
More than one adult in household	Weekly hours outdoor sleeping	0.045760	53	0.034416
Employment (working/maternity leave/unemployed)	Frequency outdoor sleeping	0.051561	54	0.035065
Preterm (yes/no)	Frequency during weekdays	0.051862	55	0.035714
Gestational age at birth	Frequency carrying outdoors	0.061123	56	0.036364
Working hours weekly	Weekly hours outdoor sleeping	0.061163	57	0.037013
Gestational age at birth	Weekly hours outdoor sleeping	0.061248	58	0.037662
More than one child in household	Sleeping outdoors (Yes/No)	0.062647	59	0.038312
Employment (working/maternity leave/unemployed)	Frequency carrying outdoors	0.066534	60	0.038961
Infant behavior at night	Frequency during weekdays	0.067303	61	0.039610
More than one adult in household	Frequency outdoor sleeping	0.075047	62	0.040260
Working hours weekly	Frequency during weekdays	0.076284	63	0.040909
More than one child in household	Frequency during weekends	0.089743	64	0.041558
Infant sex	Frequency during weekends	0.108739	65	0.042208
Maternal physiological health issues (yes/no)	Frequency during weekends	0.111950	66	0.042857
House (detached/semidetached/terraced/apartment)	Frequency during weekends	0.113118	67	0.043506
Maternal mental health issues (yes/no)	Frequency during weekdays	0.115972	68	0.044156
City size	Frequency outdoor sleeping	0.117820	69	0.044805
Types of different recreational areas nearby	Frequency outdoor sleeping	0.131656	70	0.045455
House (detached/semidetached/terraced/apartment)	Frequency outdoor sleeping	0.133440	71	0.046104
Gestational age at birth	Walking weekly in minutes	0.134755	72	0.046753
Employment (working/maternity leave/unemployed)	Weekly hours outdoor sleeping	0.141557	73	0.047403
Education level (higher/lower)	Weekly hours outdoor sleeping	0.145874	74	0.048052
More than one child in household	Frequency outdoor sleeping	0.151057	75	0.048701
Infant health issues (yes/no)	Walking weekly in minutes	0.151775	76	0.049351

Maternal physiological health issues (yes/no)	Frequency carrying outdoors	0.155933	77	0.050000
More than one adult in household	Sleeping outdoors (Yes/No)	0.159985	78	0.050649
Infant age	Walking weekly in minutes	0.168341	79	0.051299
City size	Infant carrying (indoors+outdoors)	0.169832	80	0.051948
Working hours weekly	Frequency outdoor sleeping	0.169937	81	0.052597
House (detached/semidetached/terraced/apartment)	Frequency carrying outdoors	0.176059	82	0.053247
Maternal age	Walking weekly in minutes	0.178507	83	0.053896
Infant sex	Weekly hours outdoor sleeping	0.181046	84	0.054545
Infant behavior at night	Walking weekly in minutes	0.182443	85	0.055195
Maternal physiological health issues (yes/no)	Infant carrying (indoors+outdoors)	0.186183	86	0.055844
Infant health issues (yes/no)	Infant carrying (indoors+outdoors)	0.207275	87	0.056494
Infant age	Frequency carrying outdoors	0.234625	88	0.057143
Maternal physiological health issues (yes/no)	Walking weekly in minutes	0.236348	89	0.057792
Maternal age	Frequency during weekends	0.236854	90	0.058442
Gestational age at birth	Frequency outdoor sleeping	0.245822	91	0.059091
Types of different recreational areas nearby	Weekly hours outdoor sleeping	0.253751	92	0.059740
Infant enjoyment of outdoor walks	Infant carrying (indoors+outdoors)	0.254177	93	0.060390
Maternal enjoyment of outdoor walks	Frequency carrying outdoors	0.258624	94	0.061039
Infant health issues (yes/no)	Frequency during weekends	0.274817	95	0.061688
Infant health issues (yes/no)	Sleeping outdoors (Yes/No)	0.295835	96	0.062338
Gestational age at birth	Sleeping outdoors (Yes/No)	0.297608	97	0.062987
Infant age	Frequency during weekends	0.308034	98	0.063636
Infant behavior at night	Frequency outdoor sleeping	0.319681	99	0.064286
Infant behavior at night	Frequency during weekends	0.336017	100	0.064935
Infant enjoyment of outdoor walks	Frequency carrying outdoors	0.337374	101	0.065584
Maternal age	Frequency carrying outdoors	0.350415	102	0.066234
Preterm (yes/no)	Weekly hours outdoor sleeping	0.354915	103	0.066883
Infat sex	Infant carrying (indoors+outdoors)	0.357841	104	0.067532
Maternal enjoyment of outdoor walks	Infant carrying (indoors+outdoors)	0.358651	105	0.068182
Working hours weekly	Infant carrying (indoors+outdoors)	0.362114	106	0.068831
Season (spring/summer/fall/winter)	Frequency outdoor sleeping	0.372870	107	0.069481
Infant behavior at night	Weekly hours outdoor sleeping	0.382411	108	0.070130
Health issues (yes/no)	Frequency during weekdays	0.383044	109	0.070779
Maternal age	Frequency outdoor sleeping	0.407550	110	0.071429

Season (spring/summer/fall/winter)	Frequency carrying outdoors	0.431373	111	0.072078
Preterm (yes/no)	Sleeping outdoors (Yes/No)	0.437216	112	0.072727
Infat sex	Walking weekly in minutes	0.447117	113	0.073377
City size	Frequency carrying outdoors	0.451154	114	0.074026
Infant behavior at night	Sleeping outdoors (Yes/No)	0.452820	115	0.074675
Maternal age	Weekly hours outdoor sleeping	0.478592	116	0.075325
Education level (higher/lower)	Frequency outdoor sleeping	0.482907	117	0.075974
Infant sex	Frequency during weekdays	0.485194	118	0.076623
More than one adult in household	Infant carrying (indoors+outdoors)	0.493723	119	0.077273
Health issues (yes/no)	Weekly hours outdoor sleeping	0.505543	120	0.077922
Employment (working/maternity leave/unemployed)	Frequency during weekends	0.546157	121	0.078571
Gestational age at birth	Infant carrying (indoors+outdoors)	0.548223	122	0.079221
Working hours weekly	Frequency carrying outdoors	0.557721	123	0.079870
Working hours weekly	Frequency during weekends	0.571810	124	0.080519
Working hours weekly	Sleeping outdoors (Yes/No)	0.596097	125	0.081169
Infant sex	Frequency outdoor sleeping	0.604391	126	0.081818
City size	Frequency during weekends	0.615183	127	0.082468
Maternal physiological health issues (yes/no)	Weekly hours outdoor sleeping	0.633605	128	0.083117
Gestational age at birth	Frequency during weekends	0.647112	129	0.083766
Health issues (yes/no)	Frequency carrying outdoors	0.651964	130	0.084416
More than one adult in household	Frequency during weekdays	0.662234	131	0.085065
Maternal age	Infant carrying (indoors+outdoors)	0.667253	132	0.085714
Infat sex	Sleeping outdoors (Yes/No)	0.683042	133	0.086364
Preterm (yes/no)	Frequency carrying outdoors	0.697365	134	0.087013
Education level (higher/lower)	Walking weekly in minutes	0.706569	135	0.087662
Preterm (yes/no)	Frequency during weekends	0.706649	136	0.088312
Maternal mental health issues (yes/no)	Frequency outdoor sleeping	0.709640	137	0.088961
More than one adult in household	Walking weekly in minutes	0.724912	138	0.089610
More than one child in household	Weekly hours outdoor sleeping	0.737770	139	0.090260
Maternal mental health issues (yes/no)	Weekly hours outdoor sleeping	0.746191	140	0.090909
Health issues (yes/no)	Frequency outdoor sleeping	0.794312	141	0.091558
Season (spring/summer/fall/winter)	Infant carrying (indoors+outdoors)	0.826307	142	0.092208
Preterm (yes/no)	Frequency outdoor sleeping	0.829618	143	0.092857
City size	Frequency during weekdays	0.850924	144	0.093506
Maternal physiological health issues (yes/no)	Sleeping outdoors (Yes/No)	0.863361	145	0.094156
More than one adult in household	Frequency during weekends	0.865196	146	0.094805
Infant sex	Frequency carrying outdoors	0.915924	147	0.095455

Maternal mental health issues (yes/no)	Walking weekly in minutes	0.921238	148	0.096104
Preterm (yes/no)	Infant carrying (indoors+outdoors)	0.942215	149	0.096753
Maternal physiological health issues (yes/no)	Frequency outdoor sleeping	0.948564	150	0.097403
Maternal physiological health issues (yes/no)	Frequency during weekdays	0.952441	151	0.098052
Working hours weekly	Walking weekly in minutes	0.989466	152	0.098701
Maternal mental health issues (yes/no)	Frequency during weekends	0.996998	153	0.099351
Maternal mental health issues (yes/no)	Sleeping outdoors (Yes/No)	1.000000	154	0.100000

Note. Critical value = (i/m)Q; i = p-value's rank. m = total number of tests (*N* = 154). Q = false discovery rate (= 0.05).

The tests highlighted in grey were significant after the Benjamini-Hochberg correction.
